# The Influence of Prior Statin Use on the Prevalence and Exacerbation of Chronic Obstructive Pulmonary Disease in an Adult Population

**DOI:** 10.3389/fmed.2022.842948

**Published:** 2022-03-24

**Authors:** Joo-Hee Kim, Hyo Geun Choi, Mi Jung Kwon, Ji Hee Kim, Ji-Young Park, Yong Il Hwang, Seung Hun Jang, Ki-Suck Jung

**Affiliations:** ^1^Division of Pulmonary, Allergy, and Critical Care Medicine, Department of Medicine, Hallym University Sacred Heart Hospital, Hallym University College of Medicine, Anyang, South Korea; ^2^Department of Otorhinolaryngology-Head & Neck Surgery, Hallym University Sacred Heart Hospital, Hallym University College of Medicine, Anyang, South Korea; ^3^Department of Pathology, Hallym University Sacred Heart Hospital, Hallym University College of Medicine, Anyang, South Korea; ^4^Department of Neurosurgery, Hallym University Sacred Heart Hospital, Hallym University College of Medicine, Anyang, South Korea

**Keywords:** chronic obstructive pulmonary disease, hydroxymethylglutaryl-CoA reductase inhibitors, symptom flare up, prevalence, cohort studies

## Abstract

**Background:**

Statins have anti-inflammatory and antioxidant properties, and previous studies have reported the positive effects of statins on chronic obstructive pulmonary disease (COPD) outcomes. However, the effects of statins on the development and acute exacerbations of COPD remain unclear. Therefore, this study aimed to assess the relation between statin use and COPD occurrence in all participants and the link between statin use and COPD acute exacerbations in participants with COPD.

**Methods:**

This case-control study comprised 26,875 COPD participants and 107,500 control participants who were 1:4 matched from the Korean National Health Insurance Service-Health Screening Cohort. Conditional logistic regression was used to evaluate the probability of COPD occurrence associated with previous statin use. In addition, unconditional logistic regression was employed to assess the risk of exacerbations related to statin use among COPD participants. These relations were estimated in subgroup analysis according to statin type (lipophilic vs. hydrophilic).

**Results:**

The association between previous statin use and the occurrence of COPD did not reach statistical significance in the overall population (adjusted odds ratio [aOR] = 0.96, 95% confidence interval [CI] = 0.93–1.00, *P* = 0.059). However, statin use decreased the probability of exacerbations in participants with COPD (aOR = 0.79, 95% CI = 0.74–0.85, *P* < 0.001). Lipophilic statins decreased the probability of exacerbations, whereas hydrophilic statins were not associated with a decreased likelihood of exacerbations (aOR = 0.78, 95% CI = 0.72–0.84, *P* < 0.001 for lipophilic statins; aOR = 0.89, 95% CI = 0.78–1.02, *P* = 0.102 for hydrophilic statins).

**Discussion:**

Statin use was not associated with the occurrence of COPD in the adult population. However, statin use was associated with a reduced probability of exacerbations in participants with COPD, with a greater risk reduction with lipophilic statin use.

## Introduction

Chronic obstructive pulmonary disease (COPD) is a common, preventable, and treatable disease, but it has a progressive nature characterized by airflow limitation and decreased lung function (1). Acute exacerbations (AEs) of COPD are important events in COPD management because AEs can lead to hospitalization, worsening quality of life, progression of the disease, and increased mortality (2, 3). In addition, exacerbations become more frequent and more severe as the disease progresses (4). Therefore, several interventions, including smoking cessation, patient-education programs, triple therapy [combination of an inhaled glucocorticoid (ICS), a long-acting muscarinic antagonist (LAMA), and a long-acting beta-agonist (LABA)], and the addition of phosphodiesterase-4 (PDE4) inhibitors, are recommended to prevent AEs (5, 6). However, the effects of these interventions on exacerbation frequency are still limited, suggesting that further adjunctive therapies are required.

Statins are competitive inhibitors of 3-hydroxy-3-methyl glutaryl coenzyme A (HMG-CoA) reductases, which catalyze the rate-limiting step in cholesterol biosynthesis (7). Statins were reported to have anti-inflammatory and antioxidant effects in addition to their lipid-lowering properties (8). Due to these pleiotropic effects, statins have been suggested to have favorable effects in patients with COPD (9). Previous systematic reviews have reported that statins reduce the risk of mortality, hospitalization, and levels of inflammatory markers such as C-reactive protein (CRP) and interleukin-6 (IL-6) in COPD patients (10, 11). A recent randomized controlled trial (RCT) stated that one year of treatment with simvastatin at 40 mg per day decreased the risk of exacerbations (12). However, a well-known RCT, the Prospective Randomized Placebo-Controlled Trial of Simvastatin in the Prevention of COPD Exacerbations (STATCOPE), showed that simvastatin did not have any preventive effect on AEs in COPD (13). One possibility for the discrepancy between the two studies might be different characteristics between the participants. In the STATCOPE trial, participants with subclinical cardiovascular risk were excluded; however, the recent RCT by Schenk et al. enrolled those with diabetes and subclinical cardiovascular diseases. Therefore, real-world studies of COPD patients with various comorbidities need to be conducted to assess the effect of statins on AEs in COPD.

Systemic and respiratory inflammation is believed to be the major cause of lung damage in COPD (14, 15). Cigarette smoke and other exposures, such as biofuel or air pollutants, are well-established inducers of inflammation, oxidative stress, activation of inflammatory cells, and apoptosis and are suggested to be pathogenic mechanisms. These factors lead to airflow limitation and respiratory symptoms in susceptible individuals. Peak lung function in young adults and the rate of decrease in lung function are two essential factors that determine COPD susceptibility later in life (16). The VA Normative Aging Study demonstrated that statin use reduced lung function decline in the general population (17). In addition, Keddissi et al. showed that statins were associated with a slower decline in lung function in current and former smokers (18). As statins have a potent anti-inflammatory effect on airways and systems, the hypothesis that pharmacological intervention with statins can decrease the risk of COPD development needs to be confirmed. Therefore, we hypothesized that statins could prevent COPD occurrence in adults receiving statin treatment compared to those not taking statins.

This study aimed to evaluate the effect of statins on COPD by analyzing a nationwide healthcare database. The primary objective was to estimate the relation between the dates of statin prescription and occurrence of COPD in comparison with control participants. The secondary objective was to analyze the association between the dates of statin prescription and acute exacerbations in participants with COPD compared to COPD participants without acute exacerbations.

## Materials and Methods

### Data Sources

The Korean National Health Insurance Service-Health Screening Cohort (NHIS-NSC) data were used for this study; a comprehensive description of this cohort is provided elsewhere (19, 20). The ethics committee of Hallym University (2019-10-023) approved this study.

### Study Population and Design

**FIGURE 1:**
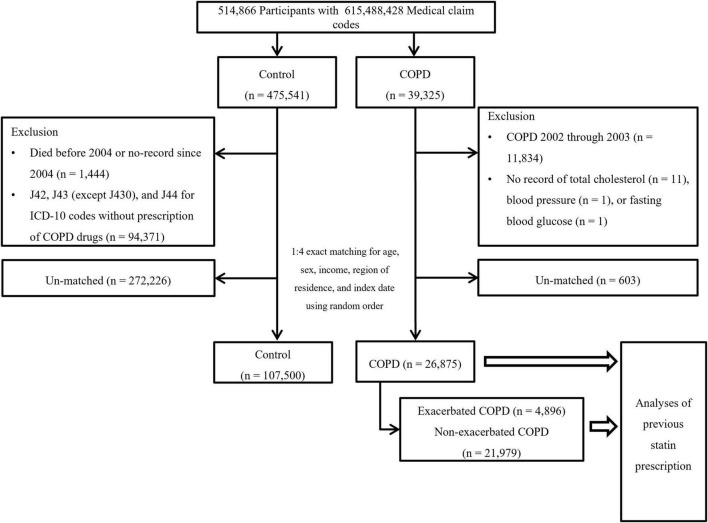
Schematic illustration of the participant selection process used in the present study. Of a total of 514,866 participants, 26,875 participants with COPD were matched with 107,500 control participants for age, sex, income, and region of residence (primary objective). The participants with COPD were classified as having exacerbated COPD (*n* = 4,896) or non-exacerbated COPD (*n* = 21,979) (secondary objective). COPD, chronic obstructive pulmonary disease.

Chronic obstructive pulmonary disease participants were selected from 514,866 participants with 615,488,428 medical claim codes (*n* = 39,325). The control group was chosen from all participants without a history of COPD during 2002–2015 (*n* = 475,541). To measure the previous 2 years of statin medication history, we excluded participants with COPD from 2002 to 2003 (*n* = 11,834). Among the control participants, we excluded 1,444 participants who died before 2004. Control participants who were diagnosed with ICD-10 codes J42, J43 (except J430), or J44 and did not have a prescription for COPD drugs during 2002–2015 were excluded (*n* = 94,371). COPD participants without records of total cholesterol (*n* = 11), blood pressure (*n* = 1), or fasting blood glucose (*n* = 1) were excluded (Figure 1). COPD participants were 1:4 matched with control participants for age, sex, income, and region of residence. To diminish the possibility of selection bias, the control participants were selected with random number order. The index date of each participant with COPD was set as the first date of the treatment, and that of the control was set by their matched COPD participant. During the matching process, 603 COPD participants and 272,226 control participants were excluded. Finally, 26,875 COPD participants were 1:4 matched with 107,500 control participants for this study. Then, previous statin use was evaluated (primary object).

The selected participants with COPD were classified into those who experienced AEs of COPD (*n* = 4,896) and those who experienced non-acute exacerbations (NAEs) of COPD (*n* = 21,979), and their history of previous statin use (secondary objective) was analyzed. In this analysis, we defined a new index date to assess the effects of statins on COPD exacerbations. For exacerbations of COPD, the first exacerbation date was set as the index date. For non-exacerbated COPD, a new random index date between the onset of COPD and the last follow-up date was chosen for fair comparison of previous statin use.

The prescription dates of statins were counted as a continuous variable for 2 years (730 days) before the index dates in COPD participants and control participants. The statins in this study included atorvastatin, fluvastatin, lovastatin, pitavastatin, pravastatin, rosuvastatin, and simvastatin. Based on pharmacological class, pravastatin and rosuvastatin were classified as hydrophilic statins, and atorvastatin, fluvastatin, lovastatin, pitavastatin, and simvastatin were categorized as lipophilic statins (21).

### Outcome Variables

Chronic obstructive pulmonary disease was defined by ≥2 occurrences of unspecified chronic bronchitis (J42), emphysema (J43), and other COPD (J44), except MacLeod syndrome (J430), as well as ≥2 prescriptions for COPD-related medications, including LAMAs, LABAs, ICSs combined with LABAs, short-acting muscarinic antagonists, short-acting beta2 agonists, methylxanthine, PDE4 inhibitors, and systemic beta agonists (22).

If participants with COPD had a history of admission or were treated/diagnosed by emergency medical doctors, they were classified as having exacerbated COPD, and the other participants with COPD were classified as having non-exacerbated COPD (23, 24).

### Covariates

Ten age groups were divided into 5-year intervals: 40–44, 45–49, 50–54,…, and 85+ years old. Income was grouped into five classes [lowest (1) through highest (5)]. The region of residence was divided into urban and rural areas following a previous study (25). Other covariates, such as smoking, alcohol consumption, and body mass index (BMI, kg/m^2^), were categorized in the same way as in a previous study (26). Blood pressure (systolic and diastolic), fasting blood glucose, and total cholesterol were measured. The Charlson Comorbidity Index (CCI) without respiratory disease was measured (0–29 score). Asthma was defined as participants who were treated for asthma (J45) or status asthmaticus (J46) following our previous study (25).

Dyslipidemia was defined if participants were treated ≥2 times for disorders of lipoprotein metabolism and other lipidemias (E78) before the index date to improve the accuracy of the diagnosis (27).

### Statistical Analyses

The demographic characteristics were compared between the COPD participants and controls and between exacerbated COPD and non-exacerbated COPD participants using standardized differences.

To estimate the odds ratios (ORs) with 95% confidence intervals (CIs) of 1 year of statin prescriptions for COPD (primary objective), conditional logistic regression was used. In this analysis, crude and adjusted models (adjusted for obesity, smoking, alcohol consumption, total cholesterol, systolic blood pressure (SBP), diastolic blood pressure (DBP), fasting blood glucose, dyslipidemia history, asthma history, and CCI score) were computed. The analysis was stratified by age, sex, income, and region of residence.

To estimate the ORs with 95% CIs for AEs (secondary objective) in those with a 1-year history of statin use, unconditional logistic regression was utilized. In this examination, crude and adjusted models were calculated using the same method as that used for the primary object.

For subgroup analyses, we divided participants by age (division point of 60 years old), sex, income, and region of residence for COPD and COPD exacerbations. We performed further subgroup analyses according to other covariates and the type of statin.

SAS version 9.4 (SAS Institute Inc., Cary, NC, United States) was utilized for the statistical analyses. Two-tailed analyses were used, and statistical significance was indicated by *P* values < 0.05.

## Results

A total of 26,875 participants with COPD and 107,500 individually matched control participants were included in this study. The general characteristics of the participants are exhibited in Table 1. Among the 26,875 COPD patients, 12.5% (*n* = 4,896) were categorized into the AE group, and the others (*n* = 21,979) were categorized into the NAE group.

**TABLE 1:** General characteristics of participants.

Characteristics	Total participants			COPD exacerbation		
	COPD	Control	Standardized difference	Exacerbated COPD	Non-exacerbated COPD	Standardized difference
Age (years), *n* (%)			0.00			0.41
40–44	302 (1.1)	1,208 (1.1)		9 (0.2)	24 (0.1)	
45–49	1,555 (5.8)	6,220 (5.8)		65 (1.3)	476 (2.2)	
50–54	2,912 (10.8)	11,648 (10.8)		207 (4.2)	1,934 (8.8)	
55–59	3,758 (14.0)	15,032 (14.0)		353 (7.2)	3,047 (13.9)	
60–64	4,429 (16.5)	17,716 (16.5)		574 (11.7)	3,375 (15.4)	
65–69	5,259 (19.6)	21,036 (19.6)		793 (16.2)	3,959 (18.0)	
70–74	4,751 (17.7)	19,004 (17.7)		1,105 (22.6)	4,168 (19.0)	
75–79	2,864 (10.7)	11,456 (10.7)		1,039 (21.2)	3,127 (14.2)	
80–84	927 (3.5)	3,708 (3.5)		583 (11.9)	1,431 (6.5)	
85 +	118 (0.4)	472 (0.4)		168 (3.4)	438 (2.0)	
Sex, *n* (%)			0.00			
Male	15,126 (56.3)	60,504 (56.3)		3,440 (70.3)	11,686 (53.2)	0.36
Female	11,749 (43.7)	46,996 (43.7)		1,456 (29.7)	10,293 (46.8)	
Income, *n* (%)			0.00			0.09
1 (lowest)	5,097 (19.0)	20,388 (19.0)		1,061 (21.7)	4,121 (18.8)	
2	3,907 (14.5)	15,628 (14.5)		709 (14.5)	2,998 (13.6)	
3	4,423 (16.5)	17,692 (16.5)		752 (15.4)	3,440 (15.7)	
4	5,615 (20.9)	22,460 (20.9)		963 (19.7)	4,515 (20.5)	
5 (highest)	7,833 (29.2)	31,332 (29.2)		1,411 (28.8)	6,905 (31.4)	
Region of residence, *n* (%)			0.00			0.21
Urban	10,201 (38.0)	40,804 (38.0)		1,423 (29.1)	8,559 (38.9)	
Rural	16,674 (62.0)	66,696 (62.0)		3,473 (70.9)	13,420 (61.1)	
Total cholesterol (mg/dL), mean (SD)	199.2 (38.6)	197.4 (39.2)	0.05	192.9 (41.1)	198.4 (38.7)	0.14
SBP (mmHg), mean (SD)	129.6 (17.9)	128.1 (17.5)	0.08	129.7 (18.4)	127.8 (17.3)	0.10
DBP (mmHg), mean (SD)	79.5 (11.1)	78.7 (10.8)	0.08	79.2 (11.2)	78.6 (10.7)	0.05
Fasting blood glucose (mg/dL), mean (SD)	102.2 (33.6)	99.8 (30.5)	0.07	100.2 (31.3)	99.8 (30.3)	0.01
Obesity^†^, *n* (%)			0.13			0.36
Underweight	1,237 (4.6)	2,777 (2.6)		487 (10.0)	750 (3.4)	
Normal	9,853 (36.7)	38,188 (35.5)		2,135 (43.6)	7,718 (35.1)	
Overweight	6,577 (24.5)	29,456 (27.4)		1,013 (20.7)	5,564 (25.3)	
Obese I	8,327 (31.0)	34,087 (31.7)		1,132 (23.1)	7,195 (32.7)	
Obese II	881 (3.3)	2,992 (2.8)		129 (2.6)	752 (3.4)	
Smoking status, *n* (%)			0.16			0.28
Non-smoker	17,741 (66.0)	77,066 (71.7)		2,711 (55.4)	15,030 (68.4)	
Past smoker	2,838 (10.6)	12,290 (11.4)		572 (11.7)	2,266 (10.3)	
Current smoker	6,296 (23.4)	18,144 (16.9)		1,613 (33.0)	4,683 (21.3)	
Alcohol consumption, *n* (%)			0.03			0.05
<1 time a week	19,169 (71.3)	75,384 (70.1)		3,396 (69.4)	15,773 (71.8)	
≥1 time a week	7,706 (28.7)	32,116 (29.9)		1,500 (30.6)	6,206 (28.2)	
Dyslipidemia, *n* (%)	6,395 (23.8)	24,119 (22.4)	0.03	851 (17.4)	5,544 (25.2)	0.19
Asthma, *n* (%)	16,322 (60.7)	16,308 (15.2)	1.06	3,642 (74.4)	12,680 (57.7)	0.36
CCI score, *n* (%)			0.18			0.58
0	15,410 (57.3)	71,046 (66.1)		1,740 (35.5)	13,670 (62.2)	
1	4,512 (16.8)	14,996 (14.0)		973 (19.9)	3,539 (16.1)	
≥2	6,953 (25.9)	21,458 (20.0)		2,183 (44.6)	4,770 (21.7)	
Date of statin prescription (day), mean (SD)	54.0 (161.4)	52.8 (161.8)	0.01	71.8 (188.5)	108.4 (226.1)	0.18
Date of hydrophilic statin prescription	9.1 (68.2)	8.7 (66.6)	0.01	14.8 (88.6)	18.8 (98.9)	0.04
Date of lipophilic statin prescription	44.9 (145.7)	44.1 (146.7)	0.01	57.0 (166.5)	89.5 (205.6)	0.17

*CCI, Charlson comorbidity index; COPD, chronic obstructive pulmonary disease; DBP, diastolic blood pressure; SBP, systolic blood pressure; SD, standard deviation.*

*^†^Obesity was categorized as a body mass index (kg/m^2^) <18.5 (underweight), ≥18.5–<23 (normal), ≥23–<25 (overweight), ≥25–<30 (obese I), and ≥30 (obese II).*

When we first examined the relation between previous statin prescription and COPD occurrence, there was no significant association in the overall population (OR = 0.96, 95% confidence interval [CI] = 0.93–1.00, *P* = 0.059 in the adjusted model, Table 2). However, subgroup analyses showed that statin prescriptions were associated with decreased incidences of the COPD diagnosis in the overweight and asthma groups (OR = 0.90, 95% CI 0.84–0.98, *P* = 0.010 for overweight and OR = 0.91, 95% CI 0.87–0.97, *P* = 0.001 for asthma, Supplementary Table 1 and Supplementary Figure 1).

**TABLE 2:** Odds ratios (95% confidence intervals) of the dates of statin prescription (1 year) for the occurrence of COPD.

Characteristics	Odds ratios for COPD			
	Crude^†^	*P*-value	Adjusted^†‡^	*P*-value
Total participants (*n* = 134,375)
Statin prescription (1 year)	1.02 (0.99–1.05)	0.267	0.96 (0.93–1.00)	0.059
Age < 60 years old (*n* = 42,635)
Statin prescription (1 year)	1.02 (1.18–0.01)	0.012*	0.88 (1.06–0.46)	0.457
Age ≥ 60 years old (*n* = 91,740)
Statin prescription (1 year)	0.97 (1.04–0.93)	0.935	0.93 (1.01–0.14)	0.140
Males (*n* = 75,630)
Statin prescription (1 year)	1.02 (0.98–1.06)	0.403	0.98 (0.93–1.04)	0.468
Females (*n* = 58,745)
Statin prescription (1 year)	1.02 (0.97–1.06)	0.463	0.94 (0.89–1.00)	0.036*
Low income (n = 67,135)
Statin prescription (1 year)	1.06 (1.01–1.11)	0.015*	0.98 (0.93–1.04)	0.537
High income (*n* = 67,240)
Statin prescription (1 year)	0.99 (0.95–1.03)	0.506	0.95 (0.90–1.00)	0.054
Urban (*n* = 51,005)
Statin prescription (1 year)	1.06 (1.01–1.10)	0.023*	0.99 (0.94–1.05)	0.815
Rural (*n* = 83,370)
Statin prescription (1 year)	0.99 (0.95–1.03)	0.619	0.94 (0.89–0.99)	0.021*

*CCI, Charlson comorbidity index; COPD, chronic obstructive pulmonary disease; DBP, diastolic blood pressure; SBP, systolic blood pressure.*

*Conditional logistic regression, significance at P < 0.05.

*^†^Models were stratified by age, sex, income, and region of residence.*

*^‡^Adjusted for total cholesterol, SBP, DBP, fasting blood glucose, obesity, smoking, alcohol consumption, history of dyslipidemia, history of asthma, and CCI scores.*

Next, we examined the effect of statins on AEs among participants with COPD. Statin prescription was associated with a decreased OR for AEs in both model 1 and model 2 (OR = 0.74, 95% CI = 0.69–0.78, *P* < 0.001 for model 1, OR = 0.79, 95% CI = 0.74–0.85, *P* < 0.001 for model 2, Table 3). Regardless of age (≥60 years), sex, income, and region of residence, statin prescription showed a significantly decreased OR for AEs in this cohort (*P* = 0.05 for all, Table 3). Subgroup analyses also showed decreased ORs in all subgroups except underweight (*P* = 0.122 for underweight, *P* < 0.05 for others, Supplementary Table 2 and Supplementary Figure 2).

**TABLE 3:** Odds ratios (95% confidence intervals) of the dates of statin prescription (1 year) for the exacerbation of COPD.

Characteristics	Odds ratios for aggravated COPD			
	Model 1^†^	*P*-value	Model 2^‡^	*P*-value
COPD participants (*n* = 26,875)		
Statin prescription (1 year)	0.74 (0.69–0.78)	<0.001*	0.79 (0.74–0.85)	<0.001*
Age < 60 years old (*n* = 6,115)
Statin prescription (1 year)	0.79 (0.64–0.96)	0.020*	0.76 (0.61–0.96)	0.018*
Age ≥ 60 years old (*n* = 20,760)
Statin prescription (1 year)	0.74 (0.69–0.78)	<0.001*	0.80 (0.74–0.85)	<0.001*
Males (*n* = 15,126)
Statin prescription (1 year)	0.75 (0.69–0.80)	<0.001*	0.83 (0.76–0.90)	<0.001*
Females (*n* = 11,749)
Statin prescription (1 year)	0.72 (0.65–0.80)	<0.001*	0.72 (0.65–0.81)	<0.001*
Low income (*n* = 13,081)
Statin prescription (1 year)	0.71 (0.65–0.78)	<0.001*	0.78 (0.71–0.86)	<0.001*
High income (*n* = 13,794)
Statin prescription (1 year)	0.64 (0.58–0.71)	<0.001*	0.80 (0.73–0.87)	<0.001*
Urban (*n* = 9,982)
Statin prescription (1 year)	0.72 (0.65–0.80)	<0.001*	0.76 (0.68–0.85)	<0.001*
Rural (*n* = 16,893)
Statin prescription (1 year)	0.75 (0.69–0.80)	<0.001*	0.81 (0.75–0.88)	<0.001*

*CCI, Charlson comorbidity index; COPD, chronic obstructive pulmonary disease; DBP, diastolic blood pressure; SBP, systolic blood pressure.*

*Unconditional logistic regression, significance at P < 0.05.

*^†^Model 1 was adjusted for age, sex, income, and region of residence.*

*^‡^Model 2 was adjusted as model 1 plus total cholesterol, SBP, DBP, fasting blood glucose, obesity, smoking, alcohol consumption, history of dyslipidemia, history of asthma, and CCI scores.*

**FIGURE 2:**
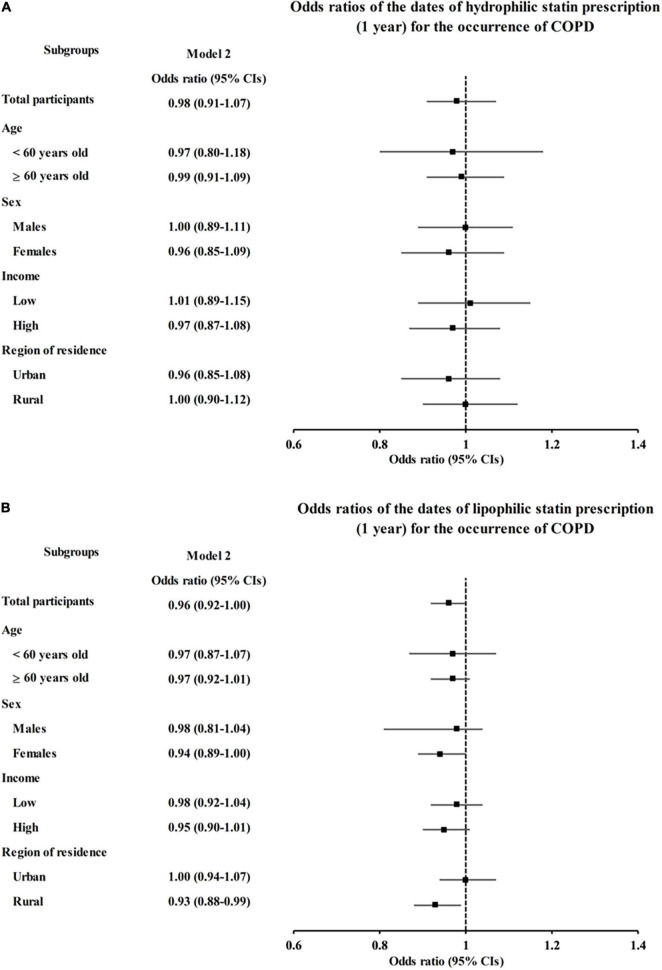
Adjusted odds ratios (95% CIs) of statin prescriptions per 1 year for COPD by subgroup: **(A)** hydrophilic statins and **(B)** lipophilic statins. Model 2 was adjusted for age, sex, region of residence, total cholesterol, SBP, DBP, fasting blood glucose, obesity, smoking, alcohol consumption, history of dyslipidemia, history of asthma, and CCI scores. CI, confidence interval; CCI, Charlson comorbidity index; COPD, chronic obstructive pulmonary disease; DBP, diastolic blood pressure; SBP, systolic blood pressure.

**FIGURE 3:**
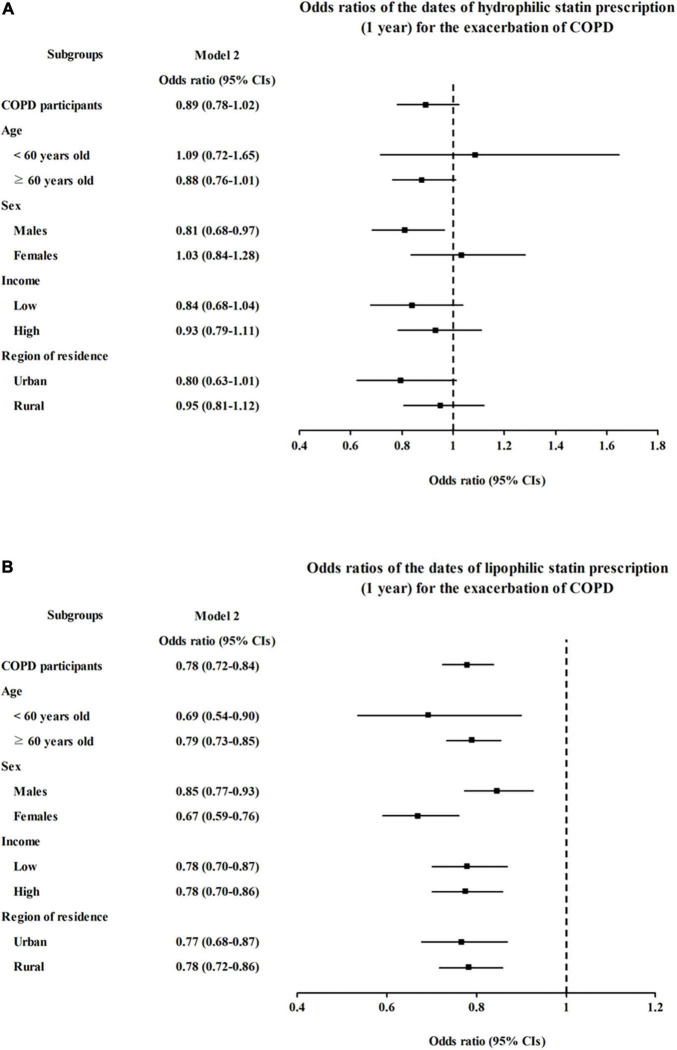
Adjusted odds ratios (95% CIs) of statin prescriptions per 1 year for COPD exacerbation by subgroup: **(A)** hydrophilic statins and **(B)** lipophilic statins. Model 2 was adjusted for age, sex, region of residence, total cholesterol, SBP, DBP, fasting blood glucose, obesity, smoking, alcohol consumption, history of dyslipidemia, history of asthma, and CCI scores. CI, confidence interval; CCI, Charlson comorbidity index; COPD, chronic obstructive pulmonary disease; DBP, diastolic blood pressure; SBP, systolic blood pressure.

We further analyzed the effect of statins on COPD diagnosis and AEs according to the pharmacological classification of statins (hydrophilic vs. lipophilic). Neither hydrophilic nor lipophilic statin prescription had any association with COPD diagnosis (OR = 0.98, 95% CI = 0.91–1.07, *P* = 0.686 for hydrophilic statins and OR = 0.96, 95% CI = 0.92–1.00, *P* = 0.065 for lipophilic statins in model 2, Figures 2A,B and Supplementary Tables 3, 4). However, hydrophilic statin prescriptions were associated with a decreased incidence of AEs in model 1 (OR = 0.83, 95% CI = 0.73–0.94, *P* = 0.005), but this association disappeared in the fully adjusted model 2 (OR = 0.89, 95% CI = 0.78–1.02, *P* = 0.102, Figure 3A and Supplementary Table 5). Lipophilic statin prescriptions decreased the incidences of AEs in both model 1 and model 2 (OR = 0.72, 95% CI = 0.67–0.77, *P* < 0.001 for model 1 and OR = 0.78, 95% CI = 0.72–0.84, *P* < 0.001 for model 2, Figure 3B and Supplementary Table 6).

## Discussion

This population-based case-control study showed that statin use decreased the probability of emergency room (ER) visits and hospitalizations due to COPD exacerbations among participants with COPD, which was consistent with the results of previous studies (10–12, 28–31). The novelty of our study includes the demonstration of different anti-inflammatory effects of statins according to their tissue selectivity; lipophilic statins showed a more profound effect on the prevention of exacerbations than hydrophilic statins. Furthermore, we examined the relationship between previous statin use and COPD occurrence in the general population using this cohort for the first time; however, previous statin use did not have any significant association with COPD occurrence.

COPD is considered a chronic systemic inflammatory syndrome, and comorbid conditions are highly prevalent in COPD patients. In particular, cardiovascular diseases are the most common comorbidities, where systemic inflammation plays a pivotal role in both conditions (14). Statins have been prescribed for the primary prevention of atherosclerotic cardiovascular diseases because they effectively lower low-density lipoprotein (LDL) cholesterol levels. In addition, the pleiotropic effects of statins, such as stabilizing the endothelium and reducing inflammatory mediators and oxidative stress, contribute to a decrease in cardiovascular morbidity (32, 33). Decreased lung function has been linked to oxidative stress and increased inflammation, and studies have proven that statins decrease proinflammatory cytokine levels in the sera of COPD patients (28, 29) and slow lung function decline (17, 18). Therefore, this study explored whether statin use decreases COPD development in an adult population. However, in this study, previous statin use did not decrease the probability of COPD occurrence in the adult population; nonetheless, the overweight and dyslipidemia subgroups showed a small but significant decrease in COPD occurrence (aOR 0.90, 95% CI 0.94–0.98, *P* = 0.010 for overweight, aOR = 0.95, 95% CI 0.91–0.99, *P* = 0.24 for dyslipidemia, Supplementary Table 1). These results imply that the effects of statins on COPD occurrence might be confined to individuals with cardiovascular diseases or that statins play a limited role in the primary prevention of COPD in general.

Statin use significantly decreased the risk of AEs among COPD patients (OR = 0.79, 95% CI 0.74–0.85, *P* < 0.001), in accordance with previous studies. Population-based case-control studies demonstrated that statin use affects AEs in COPD patients requiring hospitalization (OR = 0.67∼0.70, and HR = 0.66) (29–31, 34). A recent RCT by Schenk et al. showed that the rate of exacerbations was 1.45 events (patient-years) in the simvastatin group and 1.9 events (patient-years) in the placebo group (incidence rate ratio = 0.77, 95% CI 0.60–0.99) (12). However, the STATCOPE trial showed that simvastatin did not influence exacerbation rates or the time to a first exacerbation in COPD patients (1.36 ± 1.61 AEs for simvastatin and 1.39 ± 1.73 AEs for controls, respectively, *P* = 0.54) (13). The discrepancies among the STATCOPE trial, the RCT by Shenk et al. and other observational studies might originate from different characteristics between the study participants, as the STATCOPE trial excluded individuals with an increased risk of recurrent AEs and higher cardiovascular comorbidities, which might be different from the real-world situation. In our subgroup analyses, statins had a greater protective effect in individuals with high total cholesterol, high blood pressure, and high fasting glucose than in those without high cholesterol, high blood pressure, or high fasting glucose (OR = 0.77 vs. OR = 0.81 for high cholesterol, OR = 0.80 vs. OR = 0.78 for hypertension, OR = 0.84 vs. OR = 0.73 for hyperglycemia, Supplementary Table 2). Our results suggested that statins might be more beneficial for individuals with COPD and underlying cardiovascular disease.

Moreover, we analyzed the effect of statins on AEs according to the pharmacological class of the statin and found that lipophilic statins such as atorvastatin, simvastatin, fluvastatin, and pitavastatin have more potent preventive effects against AEs. Two RCTs chose simvastatin because it showed pleiotropic effects in *in vitro* studies and a maximal reduction in serum CRP levels at the usual dose (20–40 mg per day) without an increase in side effects (12, 13). Network meta-analysis showed supporting evidence that fluvastatin and atorvastatin (lipophilic) had a higher cumulative probability of reducing CRP in COPD patients than rosuvastatin (hydrophilic) (97.7% for fluvastatin, 68.9% for atorvastatin, and 49.3% for rosuvastatin) (28). Subgroup analyses showed that for reducing CRP, the standardized mean difference (SMD) was significantly higher for lipophilic statins than for hydrophilic statins (-0.72 for atorvastatin, -0.54 for simvastatin, and -1.66 for fluvastatin vs. -0.36 for pravastatin and -0.57 for rosuvastatin). These data suggested that the biological effects of the type of statin should be considered for further research on statins in COPD.

There are some limitations to our study. First, we defined AEs as hospitalizations or ER visits based on claim codes for patients with COPD. Therefore, our operational definition reflects only the severe degree of AEs according to the Global Initiative for Chronic Obstructive Lung Disease guidelines (1), while a mild degree of AEs was not included in this study. Second, detailed clinical variables such as cardiovascular comorbidity, pulmonary hypertension, inflammatory markers such as CRP, and lung function data were not available from the claim code dataset; therefore, these factors could not be considered in our analyses. Third, we used prescription dates of statins to calculate the duration of statin therapies; however, this may not reflect drug compliance.

However, this study has some strengths. Although we could not find any significant relationship between previous statin use and COPD occurrence, this is the first study to report the effect of statins on COPD development using a population cohort. In the present study, we reported different biological effects on AEs according to the type of statin. While statins are equivalent in potency for reducing cholesterol, they differ in their pleiotropic impact caused by lipophilicity among the types of statins. Lipophilic statins can pass through cells by passive diffusion and are distributed in diverse tissues. In contrast, hydrophilic statins are liver-specific and need carriers for uptake; therefore, they cannot exert other pleiomorphic effects on extrahepatic tissues (35). This study proved that lipophilic statins decreased the risk of AEs in COPD, which is supported by previous mechanistic studies.

In conclusion, our study showed that statin use was related to a decreased probability of COPD exacerbations requiring ER visits or hospitalization. The protective effect of lipophilic statins against AEs was more profound than that of hydrophilic statins. However, one year of statin treatment did not affect COPD occurrence in this cohort.

## Data Availability Statement

The raw data supporting the conclusions of this article will be made available by the authors, without undue reservation.

## Ethics Statement

This study was approved by the Institutional Review Board (IRB) of Hallym University (IRB No: 2019-10-023), and the need for written informed consent was waived as all participants data were obtained in an anonymous manner. Written informed consent for participation was not required for this study in accordance with the national legislation and the institutional requirements.

## Author Contributions

HC and J-HK designed this study and drafted the manuscript. HC, MK, and JK contributed to data collection and data analysis. J-YP and YH contributed to the interpretation of the data and revised the manuscript. SJ and K-SJ contributed to the final version of the manuscript. All authors read and approved the final manuscript.

## Conflict of Interest

The authors declare that the research was conducted in the absence of any commercial or financial relationships that could be construed as a potential conflict of interest.

## Publisher’s Note

All claims expressed in this article are solely those of the authors and do not necessarily represent those of their affiliated organizations, or those of the publisher, the editors and the reviewers. Any product that may be evaluated in this article, or claim that may be made by its manufacturer, is not guaranteed or endorsed by the publisher.

## Abbreviations

AE: acute exacerbation
CCI: Charlson comorbidity index
COPD: chronic obstructive pulmonary disease
DBP: diastolic blood pressure
HMG-CoA: 3-hydroxy-3-methyl glutaryl coenzyme A
ICS: inhaled glucocorticoid
LABA: long-acting beta-agonist
LAMA: long-acting muscarinic antagonist
PDE4: phosphodiesterase-4
SBP: systolic blood pressure
OR: odds ratio
RCT: randomized controlled trial.
