# Supplementary material for: The Influence of Prior Statin Use on the Prevalence and Exacerbation of Chronic Obstructive Pulmonary Disease in an Adult Population
Source: Front Med (Lausanne). 2022 Mar 24;9:842948. doi: 10.3389/fmed.2022.842948 (PMC8987534; doi:10.3389/fmed.2022.842948)
Supplement: Supplementary file 1 [file Data_Sheet_1.pdf]

S1 Table Subgroup analyses of the dates of statin prescription (1 year) for COPD in subgroups

Characteristics	Odds ratios for COPD			
	Model 1†	P-value	Model 2‡	P-value
Obesity				
Underweight (n = 4,014)	0.84 (0.64-1.12)	0.236	0.86 (0.60-1.24)	0.418
Normal weight (n = 48,041)	1.03 (0.97-1.09)	0.360	0.97 (0.90-1.04)	0.374
Overweight (n = 36,033)	0.99 (0.93-1.05)	0.742	0.90 (0.84-0.98)	0.010*
Obese (n = 46,287)	1.07 (1.02-1.12)	0.003	1.00 (0.95-1.06)	0.947
Smoking				
Nonsmoker (n = 94,807)	1.01 (0.97-1.05)	0.615	0.95 (0.90-0.99)	0.019*
Past smoker and current smoker (n = 39,568)	1.02 (0.97-1.07)	0.496	0.99 (0.92-1.06)	0.696
Alcohol consumption				
< 1 time a week (n = 94,553)	1.02 (0.98-1.06)	0.278	0.96 (0.92-1.00)	0.064
≥ 1 time a week (n = 39,822)	1.01 (0.96-1.07)	0.691	0.97 (0.91-1.05)	0.482
Total cholesterol (mg/dL)				
< 200 (n = 71,796)	0.98 (0.95-1.02)	0.402	0.93 (0.89-0.98)	0.005*
≥ 200 to < 240 (n = 44,026)	1.11 (1.04-1.18)	0.002*	1.04 (0.96-1.13)	0.321
≥ 240 (n = 18,553)	1.02 (0.94-1.11)	0.595	0.97 (0.88-1.08)	0.623
Blood pressure (mmHg)				
SBP < 140 and DBP < 90 (n = 91,000)	1.02 (0.99-1.06)	0.250	0.95 (0.90-0.99)	0.021*
SBP ≥ 140 or DBP ≥ 90 (n = 43,375)	1.00 (0.95-1.06)	0.961	1.00 (0.93-1.07)	0.902
Fasting blood glucose (mg/dL)				
< 100 (n = 82,860)	1.05 (1.01-1.10)	0.022*	0.96 (0.91-1.02)	0.193
≥ 100 (n = 51,515)	1.01 (0.97-1.06)	0.627	0.97 (0.92-1.02)	0.236
Dyslipidemia history				

Non-dyslipidemia (n = 103,861)	1.07 (0.99-1.15)	0.081	1.07 (0.98-1.16)	0.146
Dyslipidemia (n = 30,514)	0.96 (0.93-1.00)	0.058	0.95 (0.91-0.99)	0.024*
Asthma history				
Non-asthma (n = 101,745)	1.08 (1.03-1.13)	0.002*	1.02 (0.96-1.07)	0.563
Asthma (n = 32,630)	0.87 (0.83-0.91)	<0.001*	0.91 (0.87-0.97)	0.001*
CCI score (score)				
0 (n = 86,456)	1.02 (0.97-1.06)	0.436	0.95 (0.90-1.00)	0.054
1 (n = 19,508)	0.96 (0.90-1.03)	0.249	0.93 (0.86-1.02)	0.121
≥ 2 (n = 28,411)	0.98 (0.93-1.04)	0.539	0.99 (0.92-1.06)	0.681

Abbreviations: CCI, Charlson comorbidity index; COPD, chronic obstructive pulmonary disease; DBP, diastolic blood pressure; SBP, systolic blood pressure

* Un-conditional logistic regression, Significance at P < 0.05

† Model 1 was adjusted for age, sex, income, and region of residence.

‡ Model 2 was adjusted for model 1 plus total cholesterol, SBP, DBP, fasting blood glucose, obesity, smoking, alcohol consumption, dyslipidemia history, asthma history, and CCI scores.

S2 Table Subgroup analyses of the dates of statin prescription (1 year) for exacerbation of COPD by subgroup

Characteristics	Odds ratios for aggravated COPD			
	Model 1†	P-value	Model 2‡	P-value
Obesity				
Underweight (n = 1,237)	0.65 (0.44-0.95)	0.027*	0.72 (0.48-1.09)	0.122
Normal weight (n = 9,853)	0.75 (0.67-0.83)	<0.001*	0.77 (0.69-0.87)	<0.001*
Overweight (n = 6,577)	0.85 (0.75-0.95)	0.006*	0.82 (0.72-0.93)	0.002*
Obese (n = 9,208)	0.79 (0.72-0.87)	<0.001*	0.79 (0.71-0.88)	<0.001*
Smoking				
Nonsmoker (n = 17,741)	0.73 (0.67-0.79)	<0.001*	0.77 (0.71-0.84)	<0.001*
Past smoker and current smoker (n = 9,134)	0.74 (0.67-0.81)	<0.001*	0.82 (0.74-0.91)	<0.001*
Alcohol consumption				
< 1 time a week (n = 19,169)	0.75 (0.70-0.80)	<0.001*	0.79 (0.73-0.86)	<0.001*
≥ 1 time a week (n = 7,706)	0.70 (0.63-0.79)	<0.001*	0.79 (0.69-0.90)	<0.001*
Total cholesterol (mg/dL)				
< 200 (n = 14,754)	0.74 (0.68-0.80)	<0.001*	0.81 (0.74-0.89)	<0.001*
≥ 200 to < 240 (n = 8,514)	0.74 (0.66-0.83)	<0.001*	0.75 (0.67-0.85)	<0.001*
≥ 240 (n = 3,607)	0.72 (0.62-0.83)	<0.001*	0.77 (0.66-0.90)	0.001
Blood pressure (mmHg)				
SBP < 140 and DBP < 90 (n = 18,858)	0.74 (0.69-0.80)	<0.001*	0.80 (0.73-0.87)	<0.001*
SBP ≥ 140 or DBP ≥ 90 (n = 8,017)	0.72 (0.65-0.80)	<0.001*	0.78 (0.70-0.87)	<0.001*
Fasting blood glucose (mg/dL)				
< 100 (n = 17,246)	0.79 (0.73-0.85)	<0.001*	0.84 (0.77-0.92)	<0.001*
≥ 100 (n = 9,629)	0.68 (0.62-0.74)	<0.001*	0.73 (0.66-0.81)	<0.001*
Dyslipidemia history				

Non-dyslipidemia (n = 20,480)	0.75 (0.69-0.82)	<0.001*	0.76 (0.69-0.83)	<0.001*
Dyslipidemia (n = 6,395)	0.84 (0.77-0.92)	<0.001*	0.83 (0.76-0.92)	<0.001*
Asthma history				
Non-asthma (n = 10,553)	0.73 (0.65-0.82)	<0.001*	0.78 (0.68-0.89)	<0.001*
Asthma (n = 16,322)	0.73 (0.68-0.78)	<0.001*	0.79 (0.74-0.86)	<0.001*
CCI score (score)				
0 (n = 15,410)	0.70 (0.63-0.78)	<0.001*	0.81 (0.72-0.92)	0.001*
1 (n = 4,512)	0.63 (0.55-0.71)	<0.001*	0.75 (0.66-0.87)	<0.001*
≥ 2 (n = 6,953)	0.73 (0.67-0.80)	<0.001*	0.76 (0.69-0.84)	<0.001*

Abbreviations: CCI, Charlson comorbidity index; COPD, chronic obstructive pulmonary disease; DBP, diastolic blood pressure; SBP, systolic blood pressure

* Un-conditional logistic regression, Significance at P < 0.05

† Model 1 was adjusted for age, sex, income, and region of residence.

‡ Model 2 was adjusted for model 1 plus total cholesterol, SBP, DBP, fasting blood glucose, obesity, smoking, alcohol consumption, dyslipidemia history, asthma history, and CCI scores.

S3 Table Odds ratios (95% confidence interval) of the dates of hydrophilic statin prescription (1 year) for the occurrence of COPD

Characteristics	Odds ratios for COPD			
	Crude†	P-value	Adjusted†‡	P-value
Total participants (n = 134,375)				
Statin prescription (1 year)	1.04 (0.97-1.11)	0.318	0.98 (0.91-1.07)	0.686
Age < 60 years old (n = 42,635)				
Statin prescription (1 year)	1.19 (1.01-1.41)	0.043*	0.97 (0.80-1.18)	0.777
Age ≥ 60 years old (n = 91,740)				
Statin prescription (1 year)	1.01 (0.93-1.09)	0.843	0.99 (0.91-1.09)	0.871
Males (n = 75,630)				
Statin prescription (1 year)	1.05 (0.96-1.16)	0.292	1.00 (0.89-1.11)	0.930
Females (n = 58,745)				
Statin prescription (1 year)	1.02 (0.91-1.14)	0.752	0.96 (0.85-1.09)	0.517
Low income (n = 67,135)				
Statin prescription (1 year)	1.10 (0.98-1.22)	0.104	1.01 (0.89-1.15)	0.833
High income (n = 67,240)				
Statin prescription (1 year)	1.00 (0.91-1.10)	0.969	0.97 (0.87-1.08)	0.535
Urban (n = 51,005)				
Statin prescription (1 year)	1.03 (0.92-1.14)	0.654	0.96 (0.85-1.08)	0.491
Rural (n = 83,370)				
Statin prescription (1 year)	1.05 (0.95-1.15)	0.347	1.00 (0.90-1.12)	0.963

Abbreviations: CCI, Charlson comorbidity index; COPD, chronic obstructive pulmonary disease; DBP, diastolic blood pressure; SBP, systolic blood pressure

* Conditional logistic regression, Significance at P < 0.05

† Models were stratified by age, sex, income, and region of residence.

‡ Adjusted for total cholesterol, SBP, DBP, fasting blood glucose, obesity, smoking, alcohol consumption, dyslipidemia history, asthma history, and CCI scores.

S4 Table Odds ratios (95% confidence interval) of the dates of lipophilic statin prescription (1 year) for the occurrence of COPD

Characteristics	Odds ratios for COPD			
	Crude†	P-value	Adjusted†‡	P-value
Total participants (n = 134,375)				
Statin prescription (1 year)	1.01 (0.98-1.05)	0.449	0.96 (0.92-1.00)	0.065
Age < 60 years old (n = 42,635)				
Statin prescription (1 year)	1.08 (1.00-1.17)	0.067	0.97 (0.87-1.07)	0.476
Age ≥ 60 years old (n = 91,740)				
Statin prescription (1 year)	1.00 (0.96-1.04)	0.995	0.97 (0.92-1.01)	0.134
Males (n = 75,630)				
Statin prescription (1 year)	1.01 (0.96-1.06)	0.685	0.98 (0.92-1.04)	0.440
Females (n = 58,745)				
Statin prescription (1 year)	1.02 (0.97-1.07)	0.503	0.94 (0.89-1.00)	0.055
Low income (n = 67,135)				
Statin prescription (1 year)	1.05 (1.00-1.11)	0.050*	0.98 (0.92-1.04)	0.441
High income (n = 67,240)				
Statin prescription (1 year)	0.98 (0.94-1.03)	0.470	0.95 (0.90-1.01)	0.076
Urban (n = 51,005)				
Statin prescription (1 year)	1.06 (1.01-1.12)	0.022*	1.00 (0.94-1.07)	0.940
Rural (n = 83,370)				
Statin prescription (1 year)	0.98 (0.94-1.02)	0.337	0.93 (0.88-0.99)	0.012*

Abbreviations: CCI, Charlson comorbidity index; COPD, chronic obstructive pulmonary disease; DBP, diastolic blood pressure; SBP, systolic blood pressure

* Conditional logistic regression, Significance at P < 0.05

† Models were stratified by age, sex, income, and region of residence.

‡ Adjusted for total cholesterol, SBP, DBP, fasting blood glucose, obesity, smoking, alcohol consumption, dyslipidemia history, asthma history, and CCI scores.

S5 Table Odds ratios (95% confidence interval) of the dates of hydrophilic statin prescription (1 year) for the exacerbation of COPD

Characteristics	Odds ratios for aggravated COPD			
	Model 1†	P-value	Model 2‡	P-value
COPD participants (n = 26,875)				
Statin prescription (1 year)	0.83 (0.73-0.94)	0.005*	0.89 (0.78-1.02)	0.102
Age < 60 years old (n = 6,115)				
Statin prescription (1 year)	1.11 (0.74-1.65)	0.623	1.09 (0.72-1.65)	0.694
Age ≥ 60 years old (n = 20,760)				
Statin prescription (1 year)	0.80 (0.70-0.92)	0.001*	0.88 (0.76-1.01)	0.071
Males (n = 15,126)				
Statin prescription (1 year)	0.73 (0.61-0.86)	<0.001*	0.81 (0.68-0.97)	0.019*
Females (n = 11,749)				
Statin prescription (1 year)	1.06 (0.86-1.29)	0.605	1.03 (0.84-1.28)	0.758
Low income (n = 13,081)				
Statin prescription (1 year)	0.73 (0.59-0.90)	0.003*	0.84 (0.68-1.04)	0.107
High income (n = 13,794)				
Statin prescription (1 year)	0.91 (0.77-1.07)	0.256	0.93 (0.79-1.11)	0.432
Urban (n = 9,982)				
Statin prescription (1 year)	0.75 (0.59-0.94)	0.013*	0.80 (0.63-1.01)	0.063
Rural (n = 16,893)				
Statin prescription (1 year)	0.88 (0.75-1.02)	0.096	0.95 (0.81-1.12)	0.540

Abbreviations: CCI, Charlson comorbidity index; COPD, chronic obstructive pulmonary disease; DBP, diastolic blood pressure; SBP, systolic blood pressure

* Conditional logistic regression, Significance at P < 0.05

† Model 1 was adjusted for age, sex, income, and region of residence.

‡ Model 2 was adjusted for model 1 plus total cholesterol, SBP, DBP, fasting blood glucose, obesity, smoking, alcohol consumption, dyslipidemia history, asthma history, and CCI scores.

S6 Table Odds ratios (95% confidence interval) of the dates of lipophilic statin prescription (1 year) for the exacerbation of COPD

Characteristics	Odds ratios for aggravated COPD			
	Model 1†	P-value	Model 2‡	P-value
COPD participants (n = 26,875)				
Statin prescription (1 year)	0.72 (0.67-0.77)	<0.001*	0.78 (0.72-0.84)	<0.001*
Age < 60 years old (n = 6,115)				
Statin prescription (1 year)	0.69 (0.54-0.87)	0.002*	0.69 (0.54-0.90)	0.006*
Age ≥ 60 years old (n = 20,760)				
Statin prescription (1 year)	0.68 (0.64-0.73)	<0.001*	0.79 (0.73-0.85)	<0.001*
Males (n = 15,126)				
Statin prescription (1 year)	0.76 (0.69-0.82)	<0.001*	0.85 (0.77-0.93)	<0.001*
Females (n = 11,749)				
Statin prescription (1 year)	0.66 (0.59-0.74)	<0.001*	0.67 (0.59-0.76)	<0.001*
Low income (n = 13,081)				
Statin prescription (1 year)	0.71 (0.64-0.78)	<0.001*	0.78 (0.70-0.87)	<0.001*
High income (n = 13,794)				
Statin prescription (1 year)	0.73 (0.66-0.80)	<0.001*	0.78 (0.70-0.86)	<0.001*
Urban (n = 9,982)				
Statin prescription (1 year)	0.72 (0.64-0.81)	<0.001*	0.77 (0.68-0.87)	<0.001*
Rural (n = 16,893)				
Statin prescription (1 year)	0.72 (0.66-0.78)	<0.001*	0.78 (0.72-0.86)	<0.001*

Abbreviations: CCI, Charlson comorbidity index; COPD, chronic obstructive pulmonary disease; DBP, diastolic blood pressure; SBP, systolic blood pressure

* Conditional logistic regression, Significance at P < 0.05

† Model 1 was adjusted for age, sex, income, and region of residence.

‡ Model 2 was adjusted for model 1 plus total cholesterol, SBP, DBP, fasting blood glucose, obesity, smoking, alcohol consumption, dyslipidemia history, asthma history, and CCI scores.

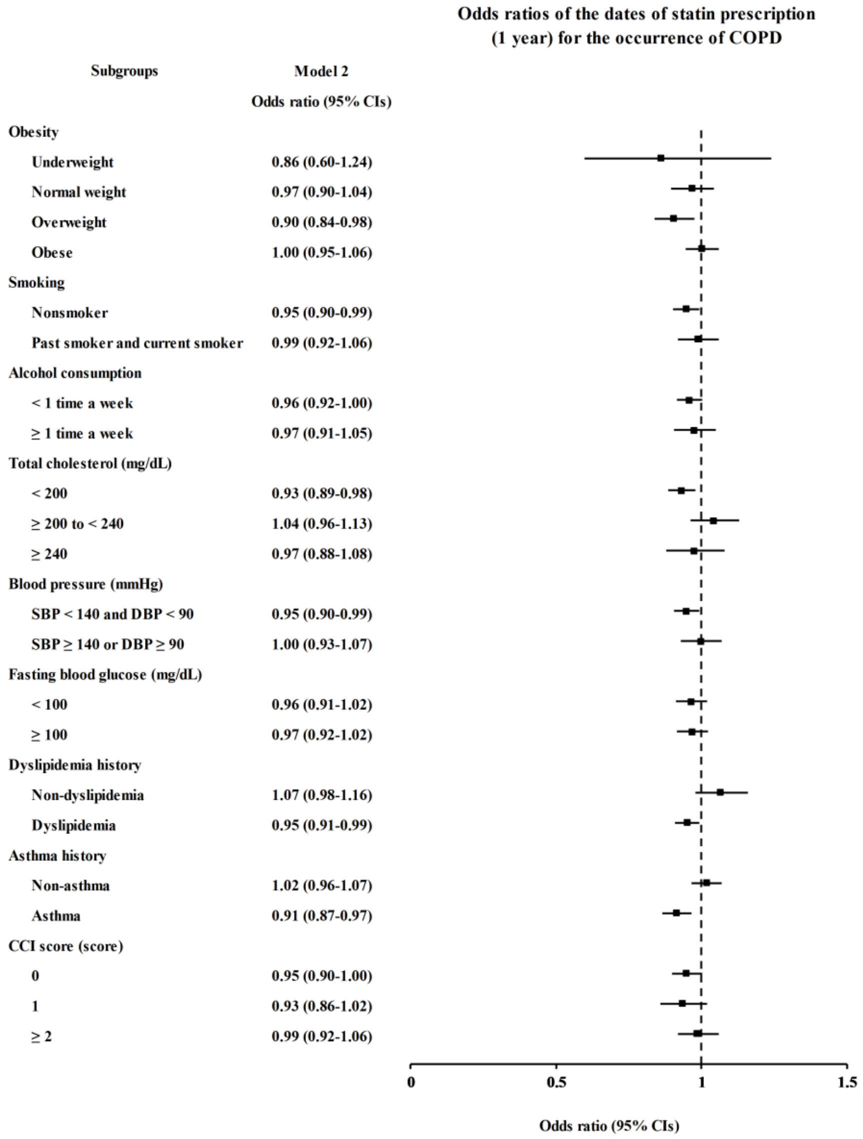

Fig. S1 Adjusted odds ratios (95% CI) of statin prescription per 1 year for the occurrence of COPD by subgroup. Model 2 was adjusted for age, sex, region of residence, total cholesterol, SBP, DBP, fasting blood glucose, obesity, smoking, alcohol consumption, dyslipidemia history, asthma history, and CCI scores.

CCI, Charlson comorbidity index; COPD, chronic obstructive pulmonary disease; DBP, diastolic blood pressure; SBP, systolic blood pressure

Odds ratios of the dates of statin prescription (1 year) for the exacerbation COPD

Subgroups	Model 2 Odds ratio (95% CIs)
**Obesity**	
Underweight	0.72 (0.48-1.09)
Normal weight	0.77 (0.69-0.87)
Overweight	0.82 (0.72-0.93)
Obese	0.79 (0.71-0.88)
**Smoking**	
Nonsmoker	0.77 (0.71-0.84)
Past smoker and current smoker	0.82 (0.74-0.91)
**Alcohol consumption**	
< 1 time a week	0.79 (0.73-0.86)
≥ 1 time a week	0.79 (0.69-0.90)
**Total cholesterol (mg/dL)**	
< 200	0.81 (0.74-0.89)
≥ 200 to < 240	0.75 (0.67-0.85)
≥ 240	0.77 (0.66-0.90)
**Blood pressure (mmHg)**	
SBP < 140 and DBP < 90	0.80 (0.73-0.87)
SBP ≥ 140 or DBP ≥ 90	0.78 (0.70-0.87)
**Fasting blood glucose (mg/dL)**	
< 100	0.84 (0.77-0.92)
≥ 100	0.73 (0.66-0.81)
**Dyslipidemia history**	
Non-dyslipidemia	0.76 (0.69-0.83)
Dyslipidemia	0.83 (0.76-0.92)
**Asthma history**	
Non-asthma	0.78 (0.68-0.89)
Asthma	0.79 (0.74-0.86)
**CCI score (score)**	
0	0.81 (0.72-0.92)
1	0.75 (0.66-0.87)
≥ 2	0.76 (0.69-0.84)

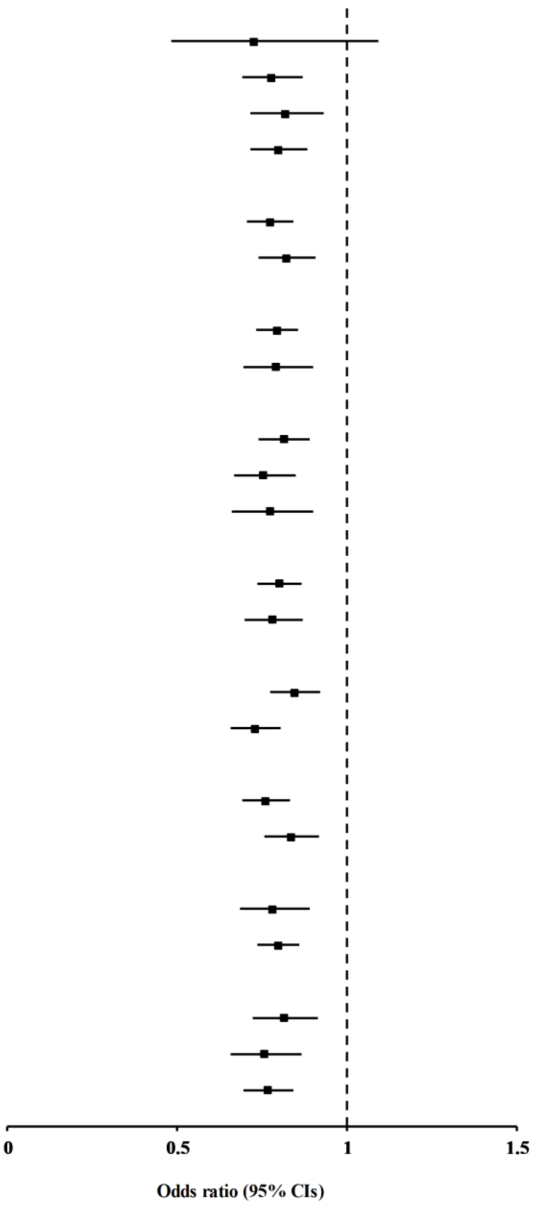

Fig. S2 Adjusted odds ratios (95% CI) of statin prescription per 1 year for the exacerbation of COPD by subgroup. Model 2 was adjusted for age, sex, region of residence, total cholesterol, SBP, DBP, fasting blood glucose, obesity, smoking, alcohol consumption, dyslipidemia history, asthma history, and CCI scores.

CCI, Charlson comorbidity index; COPD, chronic obstructive pulmonary disease; DBP, diastolic blood pressure; SBP, systolic blood pressure
